# A multiscale quantum dots-based material platform for high-performance immunosensing of rhinitis biomarkers

**DOI:** 10.1016/j.mtbio.2026.102839

**Published:** 2026-01-21

**Authors:** Jingqiu Chen, Hegeng Li, Yanbing Tao, Wenjian Zhang, Xinyi Chen, Yunong Zhao, Lanpeng Guo, Qing Huang, Jianjun Chen, Huan Liu

**Affiliations:** a School of Integrated Circuits, Huazhong University of Science and Technology, Wuhan, Hubei, PR China; b Department of Otorhinolaryngology, Union Hospital, Tongji Medical College, Huazhong University of Science and Technology, Wuhan, Hubei, PR China; c Optics Valley Laboratory, Huazhong University of Science and Technology, Wuhan, Hubei, PR China; d Wuhan National Laboratory for Optoelectronics, Huazhong University of Science and Technology, Wuhan, Hubei, PR China

**Keywords:** Immunosensors, Rhinitis diagnostic, ECP/MPO detection, Quantum dots, Multiscale materials

## Abstract

Rhinitis is a common chronic respiratory disease that severely affects patients' quality of life and may lead to serious complications, underscoring the need for rapid and portable diagnostic tools to support routine monitoring and clinical management. Here, we introduce a multiscale material platform based on colloidal quantum dots (CQDs) for the construction of electrochemical immunosensors enabling the rapid detection of eosinophil cationic protein (ECP) and neutrophil myeloperoxidase (MPO), two clinically relevant biomarkers of rhinitis. The platform leverages specific antigen–antibody recognition coupled with the electrical transduction properties of CQDs, resulting in significant improvement of detection sensitivity. To ensure translational applicability, we systematically examined key factors associated with real clinical samples, including hemolysis, storage duration, and preservation conditions in serum, and verified the method's reliability through spiked recovery experiments. More over, the immunosensor was successfully applied to the detection of ECP and MPO in patient-derived nasal secretion samples, exhibiting strong concordance with enzyme-linked immunosorbent assay (ELISA) results. Collectively, this work demonstrates that the CQD-based material platform offers a robust and clinically validated strategy for protein biomarker detection, and highlights its strong potential for home-based diagnostics, providing valuable support for the early diagnosis and management of rhinitis.

## Graphical abstract

**Figure:**
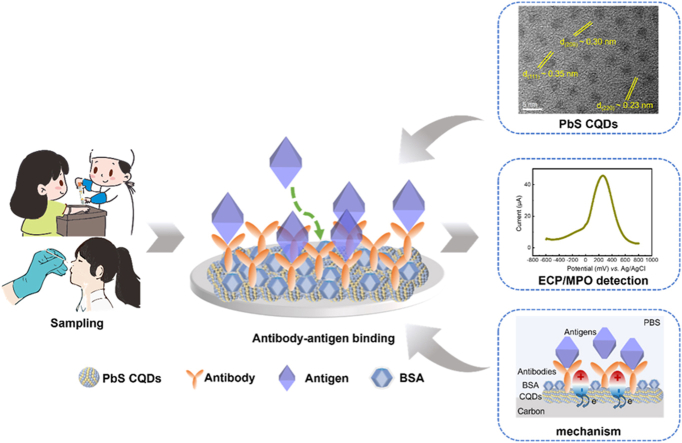

## 1 Introduction

Rhinitis is one of the most common chronic respiratory diseases, with the prevalence of allergic rhinitis (AR) reported to range from 5 % to 50 % worldwide [1,2]. Beyond significantly impairing patients’ quality of life and work productivity, rhinitis can also lead to serious complications such as sinusitis, asthma, and olfactory dysfunction [3,4]. Conventional diagnostic methods, including skin prick tests (SPT), nasal endoscopy, and imaging techniques, heavily rely on expensive equipment and complex biochemical analyses [5,6]. These limitations underscore the urgent need for rapid and portable diagnostic tools suitable for routine clinical sample analysis and disease management.

Previous research indicates that eosinophil cationic protein (ECP) and myeloperoxidase (MPO) in serum and nasal secretions are closely associated with nasal inflammation, displaying distinct expression patterns across rhinitis subtypes [[7], [8], [9]]. Elevated MPO with low ECP levels typically suggests infectious rhinitis, whereas elevated ECP with reduced MPO is indicative of allergic rhinitis. Although colloidal gold immunoassay (CGIA) strips have been used for rapid screening of ECP/MPO, they are limited to qualitative analysis [10,11]. However, quantitative monitoring of protein levels is crucial for early diagnosis and evaluation of therapeutic outcomes.

Immunosensors represent a promising approach for sensitive and quantitative detection of biomarkers, typically relying on specific antigen–antibody interactions for biomarker recognition [12,13]. However, the inherently poor electrical conductivity of proteins often limits sensor performance. To address this, various conductive nanomaterials (gold nanoparticles, graphene, or quantum dots) have been widely utilized to improve the transduction efficiency of biomolecular signals [[14], [15], [16]]. In addition, plasmon resonance and microfluidic technologies have also been incorporated into biosensors to enhance their detection performance [17,18]. Despite these advances, practical clinical application remains challenging due to several real-world factors, such as variable sample collection times, inconsistent sample storage conditions, and the presence of interfering substances in complex biological matrices, all of which can significantly affect assay accuracy and reliability [19].

**Fig. 1:**
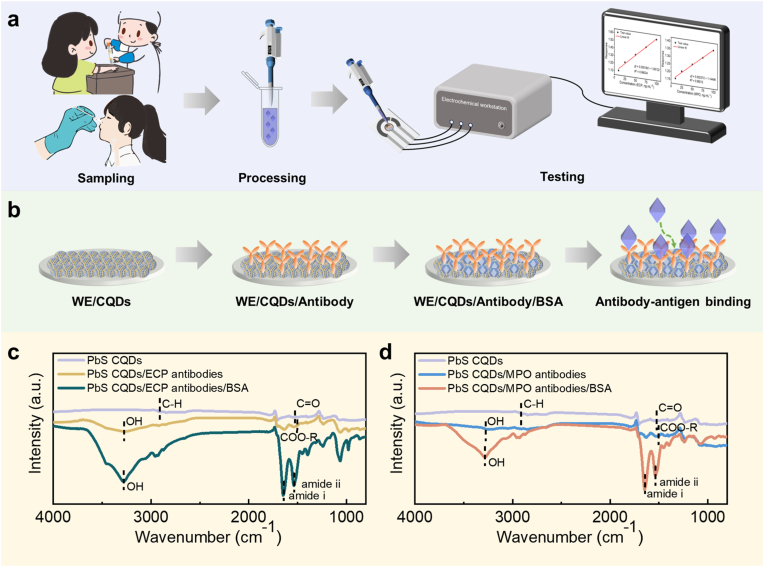
Construction and characterization of immunosensors. (a) Sample testing procedures. (b) Construction of immunosensors. (c) The FTIR spectra of PbS CQDs, PbS CQDs/ECP antibodies, PbS CQDs/ECP antibodies/BSA. (d) The FTIR spectra of PbS CQDs, PbS CQDs/MPO antibodies, PbS CQDs/MPO antibodies/BSA.

In this work, we developed immunosensors for the simultaneous detection of ECP and MPO in artificial serum and nasal secretion samples (Fig. 1(a)). The immunosensors are constructed using colloidal quantum dots (CQDs) as labels for ECP and MPO antibodies. Specific antigen–antibody binding induces a redistribution of surface potential, which can be transduced into readable electrical signals by exploiting the excellent electrical transduction properties of CQDs. Considering the particular characteristics of clinical samples, the effects of hemolysis, storage duration, and preservation conditions in serum samples were systematically optimized. The applicability of the proposed method for ECP and MPO detection in serum was further validated by spiked recovery experiments. In addition, the CQDs-based immunosensor was applied to the detection of ECP and MPO in nasal secretion samples. Comparative analysis with ELISA demonstrated the practicality of the proposed approach. This CQDs-based immunosensor strategy provides a novel method for detecting ECP and MPO in clinical samples and holds great promise for point-of-care testing (POCT) and home-based diagnostics to meet the urgent needs of rhinitis management.

## 2 Results and discussion

### 2.1 Fabrication and characterization of CQDs-based immunosensors

The prepared CQDs were drop-cast onto the working electrode of the three-electrode system to form a CQD film. Then, antibodies and BSA were sequentially drop-added. With this, the fabrication of the CQDs-based immunosensor was completed (Fig. 1(b)). The field-emission transmission electron microscopy (FTEM) images in Fig. S1(b) illustrate the morphology of PbS CQDs, revealing the spherical shape with a diameter of ∼4 nm. The UV–Vis–NIR absorption spectrum in Fig. S1(c) exhibits a prominent absorption peak at a wavenumber of 1221 nm. Employing the Moreels method, the size of PbS CQDs is calculated to be 4.2 nm using the following formula:

eq. 1 $$\begin{array}{c} \frac{1240}{\lambda }=0.41+\frac{1}{{0.0252d\;}^{2}+0.283d} \end{array}$$

d represents the size of CQDs, λ corresponds to the wavelength of absorption peak [20]. This corresponds to the results measured by FTEM. The insert of Fig. S1(c) is the particle size distribution histogram after Gaussian nonlinear fitting. The PbS CQDs exhibit an average size of 4.2 nm with a standard deviation of 0.2 nm, which proves the excellent uniformity of the synthesized CQDs. These results show that the diameter of the CQDs is significantly smaller than the exciton Bohr radius of bulk PbS (∼18 nm). This endows PbS CQDs with unique physicochemical properties [21]. Furthermore, The X-ray diffraction (XRD) spectrum (Fig. S1(d)) demonstrates that the main diffraction peaks are consistent with JCPDS No. 05–0592. These diffraction peaks correspond to the (111), (200), and (220) crystal planes of PbS CQDs crystals, which aligns with the observations from the FTEM image (Fig. S1(b)). Fig. S2 illustrates the surface morphology of the PbS CQDs-modified electrode, revealing a flat surface with regular gaps and uniformly distributed pores. The energy-dispersive X-ray spectroscop (EDS) images confirm the uniform distribution of lead (Pb) and sulfur (S) across the electrode surface, demonstrating the uniformity of the PbS CQDs modification layer.

FTIR spectrum (Fig. 1(c) and (d)) reveals distinct C-H and C=O peaks on the PbS CQDs, which are attributed to the long-chain OA ligands [22]. After antibody immobilization, the C-H stretching vibration peak of the PbS CQDs/antibodies modification layer is weakened, and the C=O peak changes, indicating that the ligands have been altered. In addition, the emergence of OH peaks and amide bands confirm the stable presence of BSA [23,24]. It can be hypothesized that antibodies are successfully labeled by PbS CQDs through ligand exchange, and the PbS CQDs/antibodies modification layer is blocked by BSA [25]. SEM images present an increase in the surface roughness of the electrode after antibodies modification (Fig. S3(a) and Fig. S4(a)). Antibodies are proteins rich in oxygen (O) atoms, with abundant nitrogen (N) atoms primarily distributed in the backbones and side chains of amino acids. The surface of PbS CQDs-modified electrode is dominated by S and Pb elements, with a negligible N. Therefore, the abundant presence of O and N on the surface of the PbS CQDs/antibodies-modified electrode directly indicates the successful immobilization of antibodies (Fig. S3 (b)–(c) and Fig. S4 (b)–(c)). Uniform distribution of O and N implies that the antibodies are homogeneously anchored on the electrode, which is crucial for consistent and stable sensor performance.

**Fig. 2:**
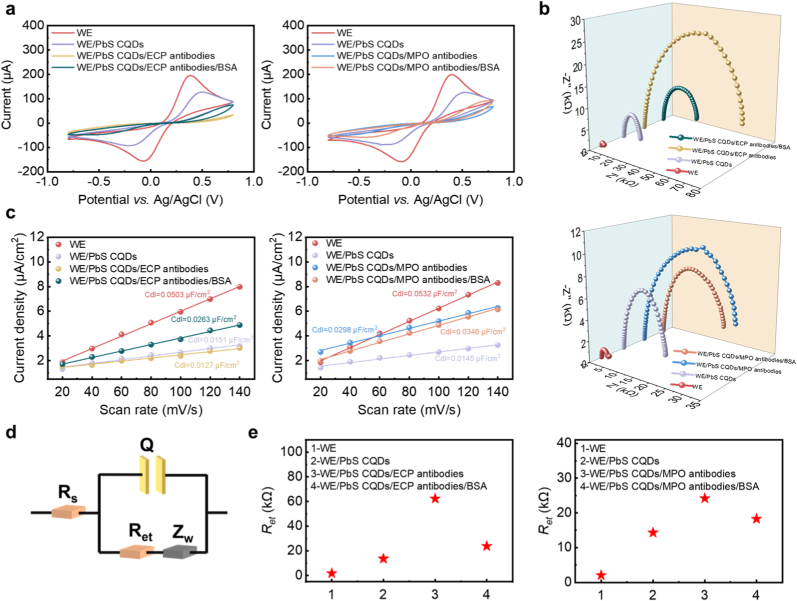
The electrical performance of the modified electrodes. (a) CV curves of electrodes with different modification layers. (b) EIS responses of the modified electrodes. (c) Relationship between current density and scan rate for the modified electrodes. (d) Equivalent circuit model established to fit the EIS data. (e) the *R*_*et*_ of different modified layers.

Electrochemical methods were employed to characterize the electrical properties of the modified electrodes. The cyclic voltammetry curves are presented in Fig. 2(a). The oxidation peak currents changed with the layer-by-layer modification: compared with the bare WE, the current of the WE/PbS CQDs electrode decreased from 194.8 μA to 126.8 μA, and the potential shifted rightward from 381 mV to 487 mV, which is speculated to the long-chain oleic acid ligands on the surface of CQDs. After modification with ECP antibodies, the intrinsic insulating property of proteins led to a significant reduction in the oxidation peak current. As a mild surfactant, BSA increased the oxidation peak current after being modified on the electrode. Electrochemical impedance spectroscopy (EIS) was used to quantitatively analyze the changes in electron transfer resistance (*R*_*et*_) caused by the layer-by-layer modification (Fig. 2(b)). Combined with the equivalent circuit in Fig. 2(d), the *R*_*et*_ of different modified layers were extracted. With the sequential modification of PbS CQDs, ECP antibodies, and BSA, the *R*_*et*_ changed from 1.83 kΩ to 13.61 kΩ, 62.36 kΩ, and 23.81 kΩ (Fig. 2(e)), respectively, which is consistent with the change in the oxidation peak current in the cyclic voltammetry curves. Similar changes in electrical properties also occurred during the construction of the MPO immunosensor. Figs. S5 and S6 display the cyclic voltammetry curves of different modified electrodes at various scan rates in the non-faradaic region (−0.65 ∼ −0.55 V). The currents at −0.6 V under different scan rates were obtained, and the corresponding double-layer capacitances (*C*_*dl*_) were calculated (Fig. 2(c)). The modification of each layer resulted in a change in the double-layer capacitance, which confirmed the successful modification of PbS CQDs, antibodies, and BSA, and also indicated that they altered the electrochemical active surface area (ECSA) of the electrode.

### 2.2 Working mechanism of CQDs-based immunosensors

**Fig. 3:**
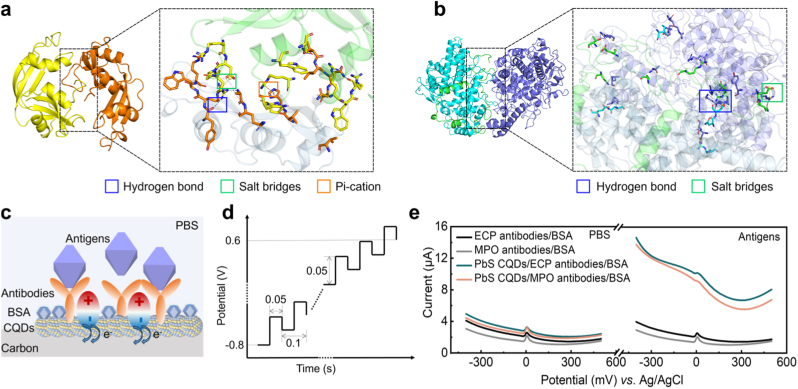
Principle of specific molecular recognition. Molecular docking simulations illustrating the binding mechanisms between antigens and antibodies: (a) ECP and (b) MPO. (c) Schematic diagram of the role of PbS CQDs in signal transduction. (d) Input voltage in DPV detection mode. (e) The DPV current curves of immunosensors with and without PbS CQDs in the PBS with and without antigens.

The HDOCK program was employed for docking simulations and investigating the interaction modes between antigens and antibodies (**Supplementary Note** 1). The optimal docking result for the ECP-ECP antibody complex yielded a *Docking_Score* of −328.78 and a *Confidence_Score* of 0.9728, indicating high reliability of the docked complex model. Fig. 3(a) displays the optimal conformer of the ECP-ECP antibody complex, with interactions including hydrogen bonds (blue dashed line), salt bridges (yellow dashed line), Pi-Cation interactions (orange dashed line), and residues involved in hydrophobic interactions. The optimal docking result for the MPO-MPO antibody complex showed a *Docking_Score* of −291.91 and a *Confidence_Score* of 0.9447. Fig. 3(b) presents the optimal conformer of the MPO-MPO antibody complex, with interactions including hydrogen bonds (blue dashed line), salt bridges (yellow dashed line), and residues involved in hydrophobic interactions. **Supplementary Notes** 2, 3 and Fig. S7 provide detailed descriptions of each type of interaction.

Since the size of CQDs is smaller than their exciton Bohr radius, confined electron/hole wavefunctions are generated and the band gap is significantly increased. A single CQD subjected to quantum confinement behaves as a dipole of an insulating capacitor. Within the specific voltage range, charge transfer from the solution to the electrode charges the CQD dipole along the CQD-antibody interface. After that, the CQD dipole will discharge at an appropriate voltage due to the coulomb blockade effect [25]. Charge transfer inside the CQDs modification layer may occur through hopping (e.g., electron sharing in bulk materials) due to wavefunction leakage or quantum tunneling. It can be speculated that under special voltage conditions, the unique charging and discharging effect of CQDs can convert the charge transfer at the CQDs-antibodies interface into the current. Following the specific binding of antigen-antibody on the electrode surface, the increase in the amount of charge brought by the antigen, combined with the charge redistribution at the antigen-antibody interface, will induce an increase in the charge at the CQDs-antibodies interface, enhancing the charge intensity of the CQD dipole and increasing the current (Fig. 3(c)).

At different antigen concentrations, the variation in the response current with temperature can be attributed to changes in the electrical properties of the CQDs modification layer and the efficiency of antigen-antibody binding. For a specific concentration, as the temperature increases, the resistance of the CQDs modification layer decreases and the charge intensity of the CQD dipoles, leading to a reduced response current. However, higher temperatures also improve the antigen-antibody binding efficiency, increasing the response current. For clinical applications, a temperature compensation module can be integrated to mitigate thermal drift.

A series of experiments were conducted to confirm the key role of CQDs in signal transduction. Differential pulse voltammetry (DPV) was used to record the response signal of the immunosensor. As shown in Fig. 3(d), a series of short pulse potentials are applied to WE, the current is measured before the start ($I_{1}$) and before the end ($I_{2}$) of each pulse, and the differential current serves as the output current ($I=I_{2}-I_{1}$). This method eliminates background current interference caused by environmental variations (such as temperature, humidity, etc.), and improves sensitivity and resolution. The output current can be fully displayed within 35 s with the parameter settings specified in this paper, highlighting its exceptional rapidity. DPV measurements were performed in PBS with and without the target proteins (10 ng mL^−1^). As shown in Fig. 3(e), no significant difference in current response of the immunosensors with and without PbS CQDs modification layer under PBS. However, in the presence of target antigens, the immunosensors with PbS CQDs modification layer exhibited larger current response than without PbS CQDs modification layer. This indicates that the CQDs modification layer converts the biological reaction generated by antigen-antibody binding into an electrical response [25,26].

### 2.3 Optimization strategy and detection for serum samples

In clinical application, the optimization of clinical sample pretreatment protocols is essential to minimize the impact of sample treatment on detection performance. Therefore, this study investigates the effects of hemolysis, placement duration at room temperature, and storage conditions on artificial serum samples, and determines the serum sample pretreatment protocol suitable for PbS CQDs-based immunosensor.

**Fig. 4:**
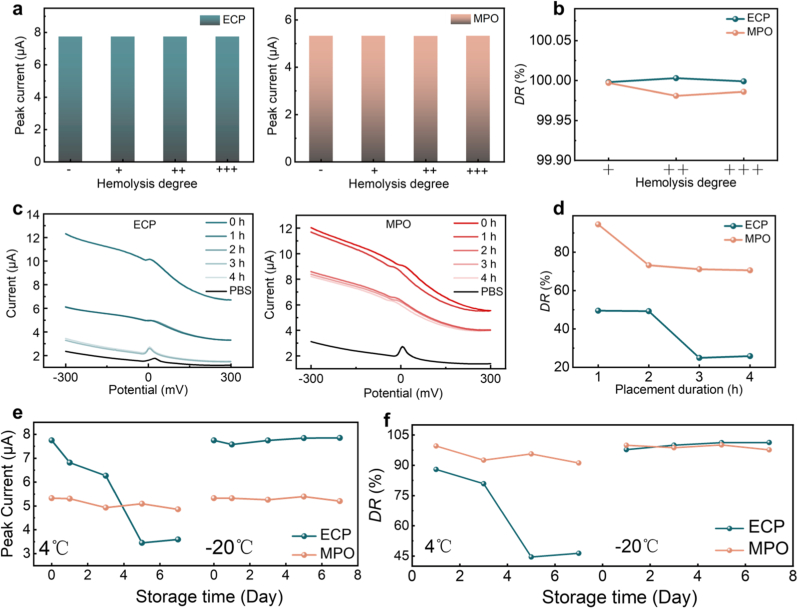
Optimization strategy for clinical samples. (a) DPV peak current with different hemolysis degree: ECP (left); MPO (right). (b) Detection rates under different hemolysis degree. (c) DPV current curves with different placement time: ECP (left); MPO (right). (d) Detection rates at different placement duration. (e) DPV peak current with different storage conditions. (f) Detection rates with different storage conditions.

To simulate different degrees of hemolysis, hemoglobin (Hgb) was added to the samples at concentrations of 0.5 g L^−1^ (mild, +), 2.5 g L^−1^ (moderate, ++), and 5.0 g L^−1^ (severe, +++), with a control sample without Hgb (−) also set up. Fig. 4(a) displays the DPV peak currents of PbS CQDs-based immunosensors for serum samples with different hemolysis conditions. And the detection rates under different hemolysis conditions were shown in Fig. 4(b) and Table S2. The results indicate that serum hemolysis has minimal impact on detection, and even severe hemolysis does not lead to significant bias.

In addition to the impact of hemolysis, the activity of biomarkers in serum samples decreases as the storage duration prolongs. In vitro diagnostics face significant challenges because the activity of biomarkers in serum samples diminishes as the placement duration increases. The in vitro half-life and instability coefficient reflect the stability of proteins. The theoretical in vitro half-lives of ECP and MPO are 1 h and 100 h, respectively, and their instability coefficient both exceed 40, indicating poor stability. To evaluate the effect of placement duration on the detection of ECP and MPO, PbS CQDs-based immunosensors were used to analyze serum samples containing 10 ng mL^−1^ of target proteins (ECP or MPO) at 26 °C. After the current curve of control group was determined (t = 0 h), analyses were performed at 1-h intervals for 4 h (Fig. 4(c)). The detection rate with different placement duration was shown in Fig. 4(d) and Table S3. The results demonstrate that the placement duration significantly impacts the detection results. For ECP, the detection rate declines to below 50 % after being placed at room temperature for 1 h and becomes almost undetectable after 3 h. MPO maintains a high detection rate within 1 h, but there is a noticeable decrease after 2 h. Therefore, for reliable quantification, ECP and MPO detection in serum samples should be completed within 1 h at room temperature.

Further exploration was conducted on the impact of serum sample storage conditions. The current of the control group was determined by detecting freshly prepared artificial serum samples containing 10 ng mL^−1^ of target proteins (ECP or MPO). According to common laboratory storage protocols, the samples were stored at 4 °C and −20 °C respectively for 7 days, with detections performed every 2 days. Prior to each detection, the frozen samples were thawed on a 37 °C heating table and subsequently returned to room temperature for 30 min before detection. The DPV peak current of the sensor is shown in Fig. 4(e). The detection rate is presented in Fig. 4(f), TableS4, and Table. S5. The results demonstrate that serum samples can maintain a high detection rate (>80 %) after being stored at 4 °C for 3 day. However, the better stability is observed at −20 °C, with no significant impact on detection rate (>97 %) for up to 1 week. Therefore, for prolonged storage periods (less than one week), serum samples should be stored at −20 °C.

### 2.4 Optimization strategy and detection of nasal secretion samples

**Fig. 5:**
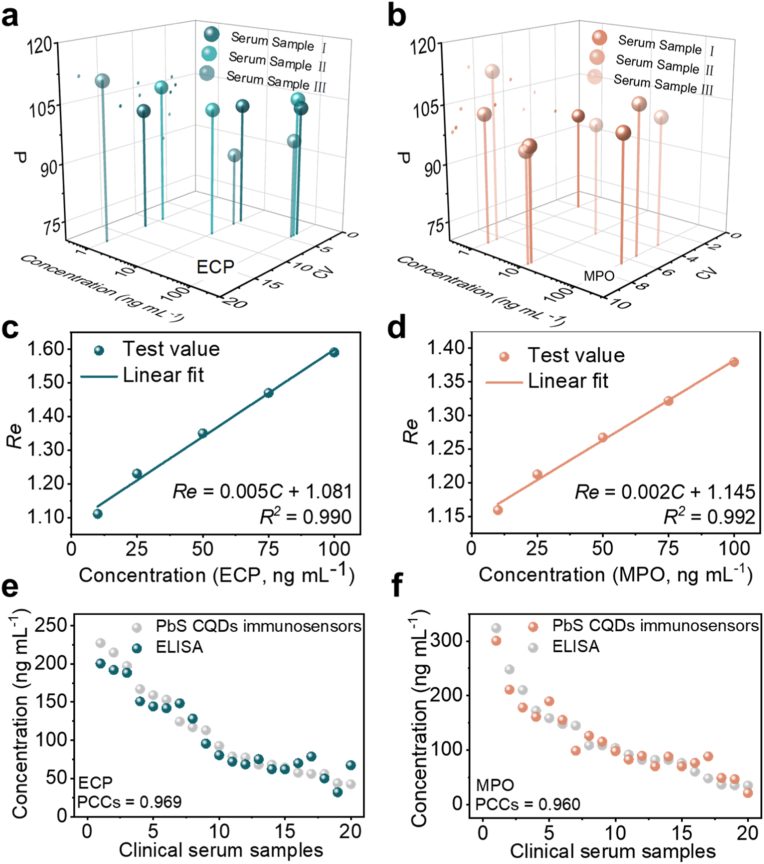
Detection in serum and nasal secretion samples. Results of spike recovery experiment in serum samples using PbS CQDs immunosensors: (a) ECP; (b) MPO. Calibration curves for PbS CQDs: (c) ECP; (d) MPO. Comparison of PbS CQDs immunosensors with ELISA: (e) ECP; (f) MPO.

A spiked recovery experiment was performed in serum to verify the practical applicability of the PbS CQDs-based immunosensors. The concentration of the target antigen in the spiked samples was deduced from the response-concentration curves (Fig. S8), and spike recovery rate (P) was calculated. The results of the spiked recovery experiment are shown in Fig. 5(a) and (b), Tables S6 and S7. For ECP detection, the P ranges from 88.90 % to 111.00 %, with a CV of 3.41 %–17.50 % (n = 3); For MPO detection, the P ranges from 96.00 % to 113.00 %, with a CV of 2.02 %–9.53 % (n = 3). These results confirm that the PbS CQDs-based immunosensors have reliable accuracy and precision in the ECP/MPO detection based on serum samples.

The detection in nasal secretions will greatly improve portability, simplify the sampling process, and enhance the accessibility of non-invasive rhinitis management. According to clinical data from the Department of Otorhinolaryngology of Wuhan Union Hospital [27], the concentration of rhinitis biomarkers in clinical nasal secretion samples is approximately 1.5–2.5 μg mL^−1^ in healthy individuals and 10–50 μg mL^−1^ in rhinitis patients. Given that the detection range of the PbS CQDs-based immunosensors is ∼ ng mL^−1^, clinical nasal secretion samples are further diluted at a ratio of 1:500, followed by extraction of the supernatant.

The calibration curves of PbS CQDs-based immunosensors based on nasal secretion samples are shown in Fig. 5(c) and (d). The calibration curve for ECP detection is expressed as Re=0.005C+1.081 and that for MPO detection is expressed as Re=0.002C+1.145. Table S1 presents the detection performance of some reported ECP/MPO sensors, among which the one proposed in this study can simultaneously meet the requirements for the detection of ECP and MPO in clinical nasal secretions.

40 nasal secretion samples (provide by the Department of Otorhinolaryngology of Wuhan Union Hospital) from rhinitis patients were analyzed using PbS CQDs-based immunosensors. Fig. 5(e) and (f) present the results of the PbS CQDs-based immunosensors and that of ELISA, the pearson correlation coefficients (PCCs) between them are 0.969 (for ECP) and 0.960 (for MPO), which confirms that the PbS CQDs-based immunosensors can quantitatively detect ECP and MPO in nasal secretion samples. Additionally, the PbS CQDs-based immunosensors display detection results within 35 s, highlighting its exceptional potential for rapid rhinitis diagnostics.

## 3 Conclusion

This study proposes protocols to rapidly and accurately detect ECP and MPO in serum and nasal secretion samples using CQDs-based immunosensors. PbS CQDs can efficiently immobilize antibodies and promote the transduction of specific antigen-antibody binding signals. From the perspective of clinical application, the effect of hemolysis, placement duration at RT, and storage conditions in serum sample were investigated and systematically optimized. The spike recovery rate ranges from 88.90 % to 113.00 %, indicating satisfactory accuracy. After dilution of nasal secretion samples, the PCCs between the test results of PbS CQDs-based immunosensors and those of ELISA reached 0.969 (ECP) / 0.960 (MPO). In addition, the single detection time is approximately 35 s. These research results demonstrate that the CQDs-based immunosensors can detect ECP and MPO rapidly, reliably, and non-invasively, highlighting their potential in point-of-care testing (POCT) and homelab for rhinitis diagnosis and management.

In the future, QDs-based immunosensors could be further developed for wearable devices, which would expand their potential applications. In situ detection of rhinitis could enable faster and more convenient diagnosis. However, several challenges need to be addressed, including the extremely high concentration of secretions and the inherently high viscosity of nasal mucus, both of which can affect measurement accuracy. Therefore, subsequent development will focus on further optimizing the sensor and readout circuit integration, with the goal of enabling convenient home-based testing.

## 4 Methods

### 4.1 Materials

The lead oxide (PbO), oleic acid (OA), octadecene (ODE), hexamethyldisilane (HMS), toluene, acetone, and n-octane, potassium ferricyanide (K_3_[Fe(CN)_6_]), potassium ferrocyanide trihydrate (K_4_[Fe(CN)_6_‧3H_2_O]), and potassium chloride were purchased from Aladdin Reagent Co., Ltd. (Shanghai, China). Bovine serum albumin (BSA) was purchased from Shanghai Aladdin Biochemical Technology Co., Ltd. (Shanghai, China). Phosphate-buffered saline (1 × PBS, PH = 7.4) was obtained from GE Healthcare Life Sciences Hyclone Laboratories (Logan, UT, USA). ECP antigens and MPO antigens were provided by AtaGenix Technology Co., Ltd. (Wuhan, China). The serum was an artificial simulated serum (BZ281, Biochemzone company). Nasal secretion was provided from Union Hospital of Tongji Medical College, Huazhong University of Science and Technology (Wuhan, China). This study was approved by the Medical Ethics Committee of Union Hospital, Tongji Medical College, Huazhong University of Science and Technology (Approval No.: 2024-0151).ECP antibodies and MPO antibodies were mouse anti-human monoclonal antibodies, provided by CHINALLERGY Biotechnology Co., Ltd. (Wuhan, China). Monoclonal antibodies can specifically bind to a single epitope of the antigen, reducing cross-reactivity with other proteins and ensuring the selectivity [28].

Serum samples containing 10 ng mL^−1^ of antigens (ECP or MPO) were prepared to analyze and optimize the effects of hemolysis, placement duration at RT, and storage conditions on detection. Three types of serum samples were prepared to analyze the detection accuracy of the PbS CQDs-based immunosensors: Serum sample I (10 ng mL^−1^ ECP, 0 ng mL^−1^ MPO), Serum sample II (0 ng mL^−1^ ECP, 10 ng mL^−1^ MPO), and Serum sample III (10 ng mL^−1^ ECP, 10 ng mL^−1^ MPO). The concentration of the analytes was determined by the standard addition method, where the spiking levels (*T*) were 0 ng mL^−1^, 1 ng mL^−1^, 10 ng mL^−1^, and 100 ng mL^−1^, respectively.

The nasal secretion sampling procedure is as follows: A sterile sponge is insert into the patient's nasal cavity and removed after absorbing sufficient secretions (1–5 min); A 2 mL syringe is used to squeeze the sponge to extract the original nasal secretions; The original nasal secretions are diluted with PBS at a ratio of 1:2000 and stored at −20 °C as clinical nasal secretion samples.

Three nasal secretion samples with undetectable ECP and MPO were mixed, and the supernatant was extracted to serve as the blank solution. Subsequently, standard solutions with ECP/MPO at concentrations of 10, 25, 50, 75, and 100 ng mL^−1^ were prepared using the blank solution to construct the calibration curves of the CQDs-based immunosensor.

### 4.2 Synthesis of CQDs

PbS CQDs are synthesized using hot injection, and the synthesis steps can be referred to the literature [29]. 1.8g PbO, 6 mL OA, and 20 mL ODE are stirred at high speed under vacuum at 90 °C for 8 h to prepare the lead precursor. The temperature of the lead precursor is then raised to 120 °C, and a mixture of 280 μL of HMS and 10 mL of ODE is injected into the lead precursor under a nitrogen atmosphere. After 30-s reaction, the reactants are rapidly cooled in an ice bath (Fig. S1(a)). The products are washed three times with toluene and acetone. Finally, the synthesized PbS CQDs are dissolved in n-octane to form a solution with a concentration of 10 mg mL^−1^.

### 4.3 Fabrication of CQDs-based immunosensors

The three-electrode was purchased by Qingdao Poten Technology Co., Ltd. (Qingdao, China), and the working electrode (WE) and counter electrode (CE) were made from carbon, while the reference electrode (RE) was made from silver/silver chloride.

The construction process of PbS CQDs-based immunosensors as following: 1 μL of PbS CQDs (10 mg mL^−1^) is dropped onto the WE of the three-electrode, forming a PbS CQDs modification layer after drying. Next, 2.5 μL of PBS containing antibodies (2.39 mg mL^−1^ for ECP antibodies and 3.96 mg mL^−1^ for MPO antibodies) is dropped onto the PbS CQDs modification layer and incubated at 37 °C for 1 h to facilitate antibody modification. Finally, 200 μL of PBS solution containing BSA (10 mg mL^−1^) is added and incubated at RT for 1 h to block the non-specific active sites [30]. After that, the electrode was rinsed with PBS and dried at RT.

### 4.4 Characterization and equipment

The morphology, size, and crystal structure of the CQDs were characterized using field emission transmission electronic microscope (FTEM), Ultraviolet–visible–near infrared (UV–vis–NIR), and X-ray Diffraction (XRD). The FTEM images were obtained from Tecnai G2 F30 (FEI, Netherlands). UV–vis–NIR absorption spectra were obtained from the SolidSpec-3700 spectrophotometer (Shimadzu, Japan). XRD patterns were acquired from x'pert3 powder X-ray diffractometer (PANalytical B.V., Netherlands). Scanning electron microscope (SEM) images and energy dispersive spectroscopy (EDS) spectra were acquired from Nova NanoSEM 450 (FEI, Netherlands) for the analysis of CQDs-modified electrodes. Fourier transform infrared spectra (FTIR) were obtained from Nicolet iS50R (Thermo Scientific, America) to characterize the CQDs-based immunosensors modification process. All electrochemical tests were carried out on the electrochemical workstation (CHI760E, Shanghai Chenhua, Shanghai, China).

### 4.5 Testing methods

The detection performance of PbS CQDs-based immunosensors is evaluated using DPV. The DPV is conducted with a starting potential of −0.8 V and an ending potential of 0.6 V. The pulse amplitude and pulse width are set to 0.05 V and 0.05 s, respectively. The pulse period is maintained at 0.1 s. The sensitivity of the measurement was configured to 10^−6^ A/V. The cyclic voltammetry is conducted with a starting potential of −0.8 V and an ending potential of +0.8 V, with a scan rate of 0.1V/s. The EIS is conducted from 0.1 Hz to 10^6^ Hz. All cyclic voltammetry and EIS are test in the 5 mM K_3_[Fe(CN)_6_]/K_4_[Fe(CN)_6_] solution.

The detection rate of the CQDs-based immunosensors is defined as:

eq.2 $$\begin{array}{c} DR=\frac{I_{\text{experimental}}}{I_{\text{control}}}\times 100\% \end{array}$$

$I_{\text{experimental}}$ and $I_{\text{control}}$ correspond to the DPV peak current of experimental group and control group, respectively. The ELISA test results are provided by CHINALLERGY Biotechnology Co., Ltd. (Wuhan, China).

The detection accuracy of serum samples was verified using a spiked recovery experiment, and quantitatively evaluated using the P. For electrochemical trace element analysis, a larger P indicates higher detection accuracy, and the acceptable range is usually 70–130 %. The calculation formula of P is as follows:

eq.3 $$\begin{array}{c} P=\left[\;\frac{C_{spiked\neq 0}-C_{piked=0}}{T}\;\right]\times 100\%\; \end{array}$$

$C_{spiked\neq 0}$ represents the detected concentration of the sample with a non-zero spiking levels minus the known concentration of the target substance in the sample; $C_{piked=0}$ represents the detected concentration of the sample with zero spiking levels minus the known concentration of the target substance in the sample; and T is the spiking levels. The coefficient of Variation (CV) was determined to evaluate the degree of data dispersion. The formula of CV is provided below:

eq.4 $$\begin{array}{c} \text{CV}=\frac{SD}{V_{0}}\times 100\% \end{array}$$

SD represents the standard deviation, and the $V_{0}$ is the mean (n = 3). A smaller the CV indicates more concentrated data, reflecting better repeatability.

For the detection of nasal secretion samples, the response of the CQDs-based immunosensors is defined as:

eq.5 $$\begin{array}{c} Re=\frac{I_{standard}}{I_{blank}} \end{array}$$

where $I_{standard}$ represents the DPV peak current of the standard solution, and $I_{blank}$ represents the DPV peak current of the blank sample.

## CRediT authorship contribution statement

**Jingqiu Chen:** Writing – review & editing, Writing – original draft, Investigation, Data curation. **Hegeng Li:** Writing – review & editing, Validation. **Yanbing Tao:** Writing – original draft, Data curation, Conceptualization. **Wenjian Zhang:** Investigation, Conceptualization. **Xinyi Chen:** Writing – review & editing, Methodology. **Yunong Zhao:** Writing – original draft, Supervision. **Lanpeng Guo:** Writing – review & editing, Validation. **Qing Huang:** Validation, Conceptualization. **Jianjun Chen:** Writing – review & editing, Funding acquisition. **Huan Liu:** Writing – review & editing, Funding acquisition, Conceptualization.

## Declaration of competing interest

The authors declare no competing interests.

## Data Availability

Data will be made available on request.
