# Supplementary material for: A multiscale quantum dots-based material platform for high-performance immunosensing of rhinitis biomarkers
Source: Mater Today Bio. 2026 Jan 21;37:102839. doi: 10.1016/j.mtbio.2026.102839 (PMC12874439; doi:10.1016/j.mtbio.2026.102839)
Supplement: Multimedia component 1 [file mmc1.docx]

A multiscale Quantum Dots-Based Material Platform for High-Performance Immunosensing of Rhinitis Biomarkers

Jingqiu Chen^1, †^, Hegeng Li^1, †^，Yanbing Tao^1, †^, Wenjian Zhang^1^, Xinyi Chen^1^, Yunong Zhao^1^, Lanpeng Guo^1^, Qing Huang^1^, Jianjun Chen^2, *^, Huan Liu^1,3,4,*^

1. School of Integrated Circuits, Huazhong University of Science and Technology, Wuhan, Hubei, P. R. China
2. Department of Otorhinolaryngology, Union Hospital, Tongji Medical College, Huazhong University of Science and Technology, Wuhan, Hubei, P. R. China
3. Optics Valley Laboratory, Huazhong University of Science and Technology, Wuhan, Hubei, P. R. China
4. Wuhan National Laboratory for Optoelectronics, Huazhong University of Science and Technology, Wuhan, Hubei, P. R. China

^*^ Corresponding author: [cjj131419@hust.edu.cn](mailto:cjj131419@hust.edu.cn) (Jianjun Chen);

[huan@hust.edu.cn](mailto:huan@hust.edu.cn) (Huan Liu).

^†^ These authors contributed equally to this work.

**Supplementary Note 1.** Antigen-antibody Docking Method

To investigate the binding regions and interaction between proteins, the professional HDOCK[1] program, which is used for protein-protein docking, was employed for docking simulations. The structure with the highest docking score was selected as the reference model for subsequent interaction analysis. The docking score was calculated based on the iterative scoring functions ITScorePP or ITScorePR. A more negative docking score indicates a higher probability of binding.

A docking score-dependent confidence score was empirically defined to represent the binding probability of the two molecules, as shown below:

$$Confindence\_score= 1.0/[1.0+e^{0.02*\left( Docking\_Score+150 \right)}]$$

A docking score-dependent confidence score was empirically defined to represent the binding probability of the two molecules, as shown below:

Roughly, when the confidence score is above 0.7, the two molecules would be very likely to bind. Interaction analysis of the docked complexes was performed using PLIP[2], and visualization was carried out using PyMOL.

[1] Y. Yan, H. Tao, J. He, S.Y. Huang, The HDOCK server for integrated protein-protein docking, Nat Protoc 15 (2020) 1829-1852.

[2] P. Schake, S.N. Bolz, K. Linnemann, M. Schroeder, PLIP 2025: introducing protein–protein interactions to the protein–ligand interaction profiler, Nucleic Acids Research 53 (2025) W463-W465.

**Supplementary Note 2.** Interaction between ECP and ECP antibody

**Hydrogen bond interactions**: A hydrogen bond was formed between ARG117 of the ECP and ALA27 of the ECP antibody (anti-ECP), with a bond length of 3.1 Å; a hydrogen bond was formed between ARG117 of the ECP and TYR60 of the anti-ECP, with a bond length of 2.7 Å; a hydrogen bond was formed between ARG114 of the ECP and ARG63 of the anti-ECP, with a bond length of 3.4 Å; hydrogen bonds were formed between GLN14 of the ECP and THR94 of the anti-ECP, with bond lengths of 3.0 Å and 3.5 Å; a hydrogen bond was formed between ARG34 of the ECP and ASN96 of the anti-ECP, with a bond length of 2.7 Å; a hydrogen bond was formed between ARG36 of the ECP and ASN96 of the anti-ECP, with a bond length of 3.4 Å.

**Salt bridge interaction**: A salt bridge was formed between ASP115 of the ECP and ARG61 of the anti-ECP, with an interaction distance of 5.2 Å.

**Pi-cation interaction**: A pi-cation interaction was formed between ARG1 of the ECP and HIS155 of the anti-ECP, with an interaction distance of 4.9 Å.

**Residues involved in hydrophobic interactions**: PRO3, GLN4, TRP10, TRP35, PRO116, and ARG117 in the ECP; ALA27, ARG28, TRP62, ARG63, LEU95, ASN97, VAL151, and VAL154 in the anti-ECP.

**Supplementary Note 3.** Interaction between MPO and MPO antibody

**Hydrogen bond interactions**: A hydrogen bond was formed between GLN4 of the MPO chain A and ARG378 of the MPO antibody (anti-MPO), with a bond length of 2.2 Å; hydrogen bonds were formed between ARG18 of the MPO chain A and ARG575 of the anti-MPO, with bond lengths of 3.2 Å, 2.0 Å, and 2.9 Å; a hydrogen bond was formed between ARG18 of the MPO chain A and LYS576 of the anti-MPO, with a bond length of 3.4 Å; a hydrogen bond was formed between ARG27 of the MPO chain A and ARG509 of the anti-MPO, with a bond length of 3.4 Å; hydrogen bonds were formed between THR48 of the MPO chain A and ARG41 of the anti-MPO, with bond lengths of 2.3 Å and 2.6 Å; a hydrogen bond was formed between ASN54 of the MPO chain A and LEU49 of the antibody, with a bond length of 3.4 Å; a hydrogen bond was formed between ARG314 of the MPO chain C and SER390 of the anti-MPO, with a bond length of 2.9 Å; a hydrogen bond was formed between ASN317 of the MPO chain C and SER390 of the anti-MPO, with a bond length of 2.9 Å; a hydrogen bond was formed between ASP318 of the MPO chain C and ARG575 of the anti-MPO, with a bond length of 2.5 Å; a hydrogen bond was formed between GLU446 of the MPO chain C and ALA52 of the anti-MPO, with a bond length of 3.1 Å.

**Salt bridge interaction**: A salt bridge was formed between GLU3 of the MPO chain A protein and ARG378 of the anti-MPO, with an interaction distance of 4.9 Å; a salt bridge was formed between GLU38 of the MPO chain A protein and ARG41 of the anti-MPO, with an interaction distance of 4.2 Å; a salt bridge was formed between ARG314 of the MPO chain C and ASP389 of the anti-MPO, with an interaction distance of 3.5 Å; a salt bridge was formed between ASP318 of the MPO chain C and ARG575 of the anti-MPO, with an interaction distance of 0.9 Å.

**Residues involved in hydrophobic interactions**: ARG18 and PRO34 of chain A, ILE158, ASN434, and GLU446 of chain C in the MPO; TYR380, THR572, ILE231, ARG124, TYR116, PRO51, and ALA52 in the anti-MPO.


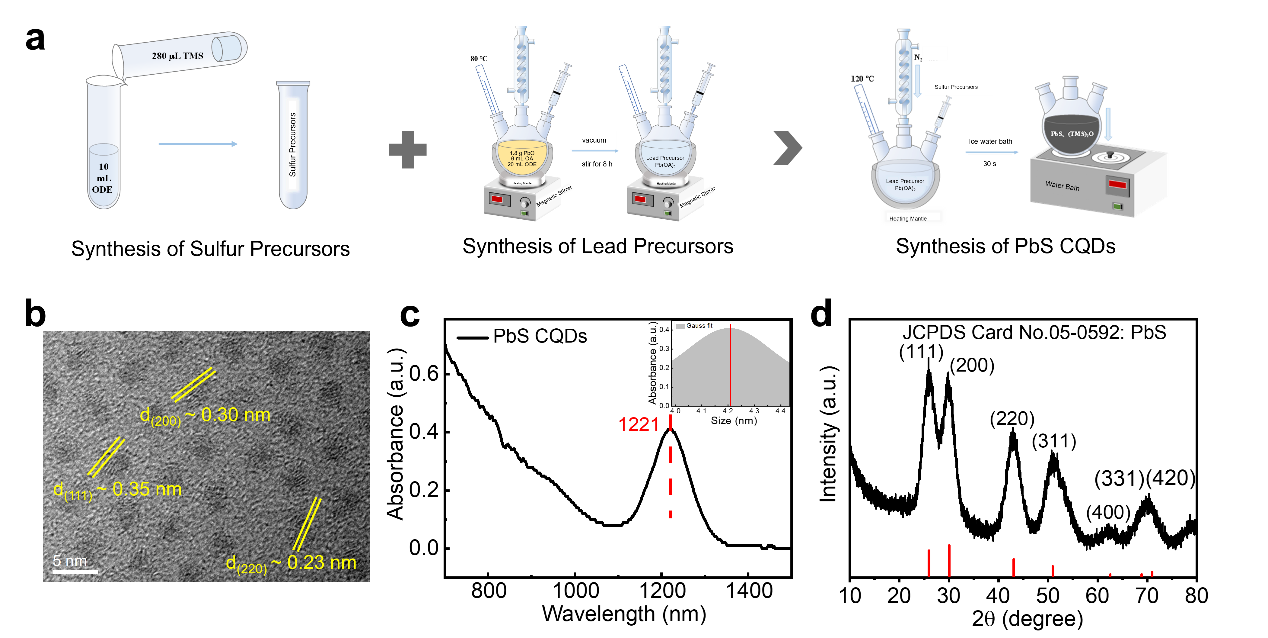


**Fig.** S1 Synthsis and Characterization of PbS CQDs. (a) Synthesis of PbS CQDs. (b) FTEM image of PbS CQDs. (c) UV–Vis–NIR absorption spectrum of PbS CQDs (The inset shows the size distribution of PbS CQDs). (d) XRD spectrum of PbS CQDs.


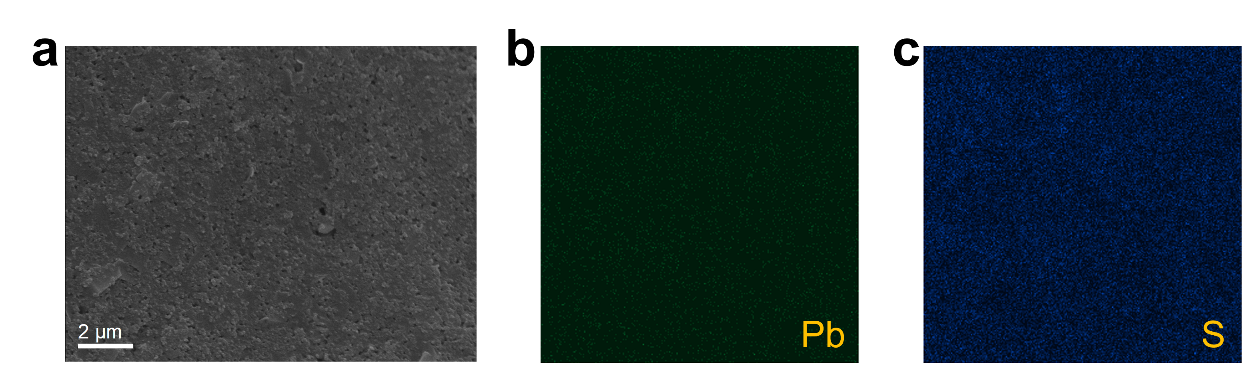


**Fig.** S2 Morphology and elemental analysis of the PbS CQDs-modified electrode. (a) SEM image of PbS CQDs-modified electrode. (b-c) Element distribution of PbS CQDs-modified electrode: (b) Pb element. (c) S element.


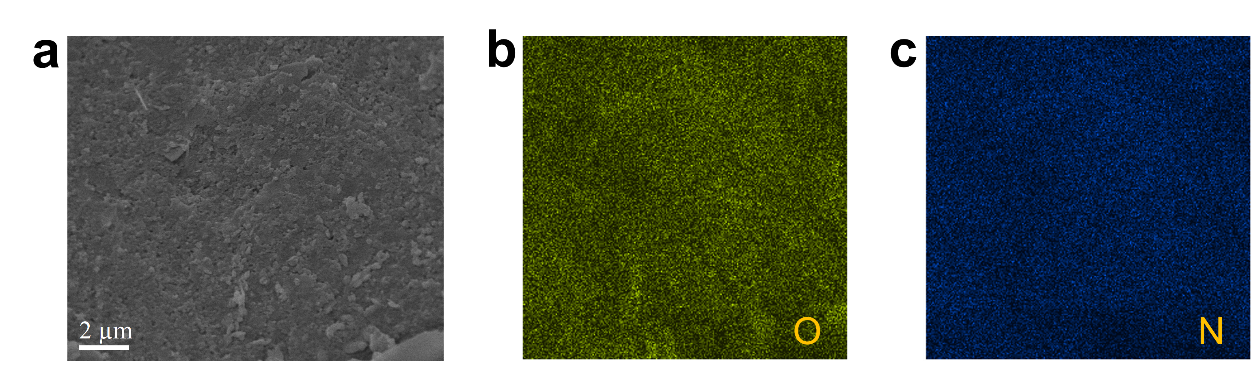


**Fig.** S3 Morphology and elemental analysis of the PbS CQDs/ECP antibodies-modified electrode. (a) SEM image of PbS CQDs/ECP antibodies-modified electrode. (b-c) Element distribution of PbS CQDs/ECP antibodies-modified electrode: (b) O element. (c) N element.


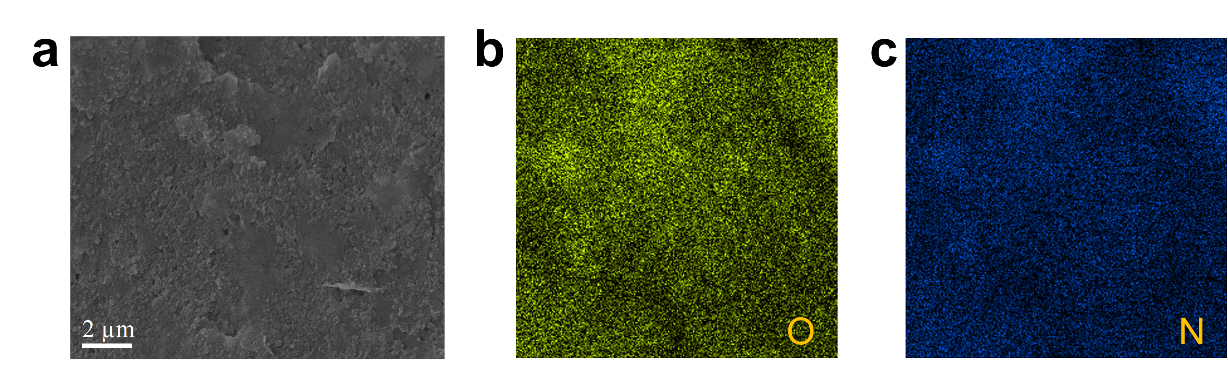


**Fig.** S4 Morphology and elemental analysis of the PbS CQDs/MPO antibodies-modified electrode. (a) SEM image of PbS CQDs/MPO antibodies-modified electrode. (b-c) Element distribution of PbS CQDs/MPO antibodies-modified electrode: (b) O element. (c) N element.


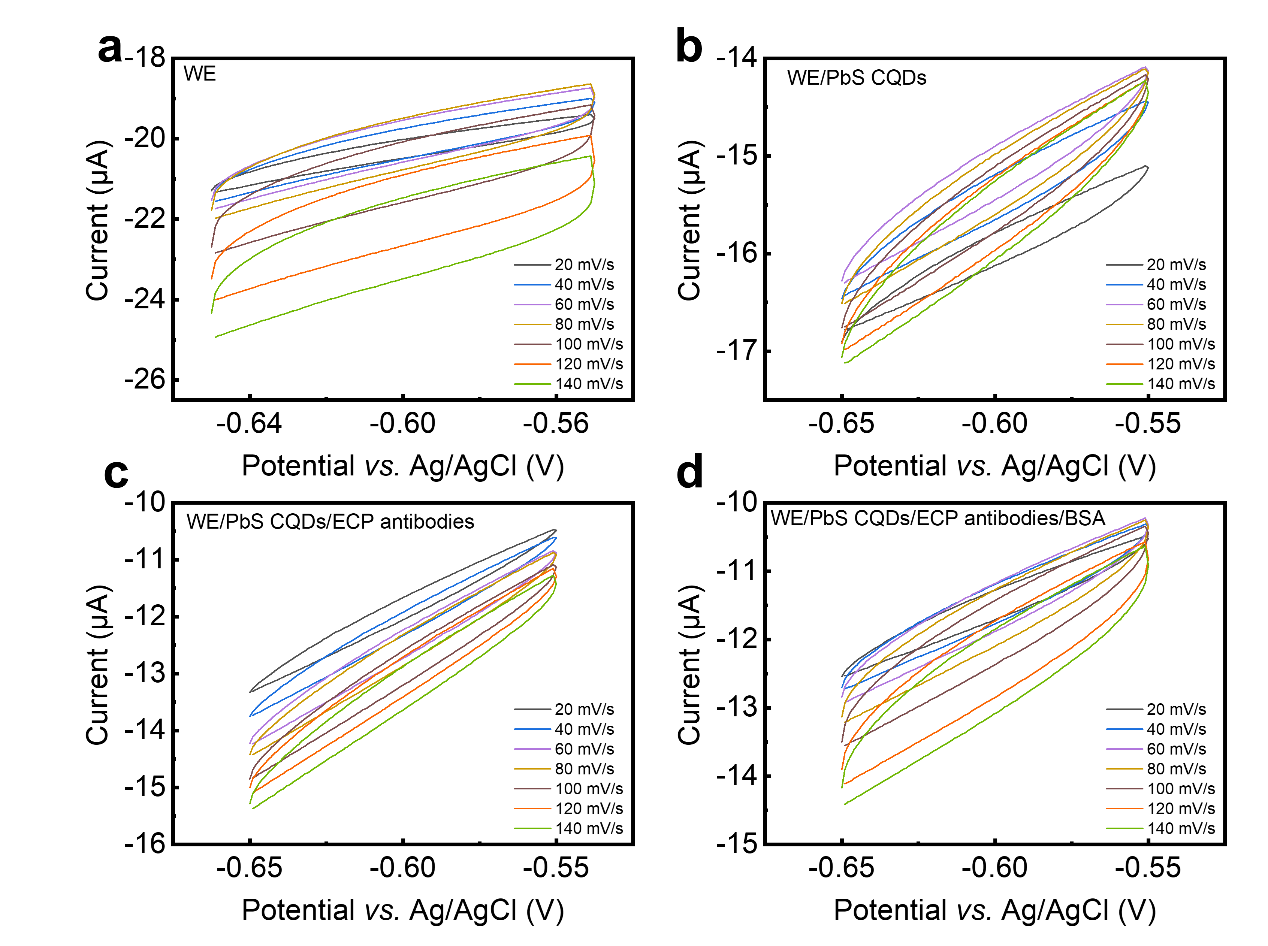


**Fig.** S5 The cyclic voltammetry curves of different modified electrodes at various scan rates in the non-faradaic region: (a) WE; (b) WE/PbS CQDs; (c) WE/PbS CQDs/ECP antibodies; (d) WE/PbS CQDs/ECP antibodies/BSA.


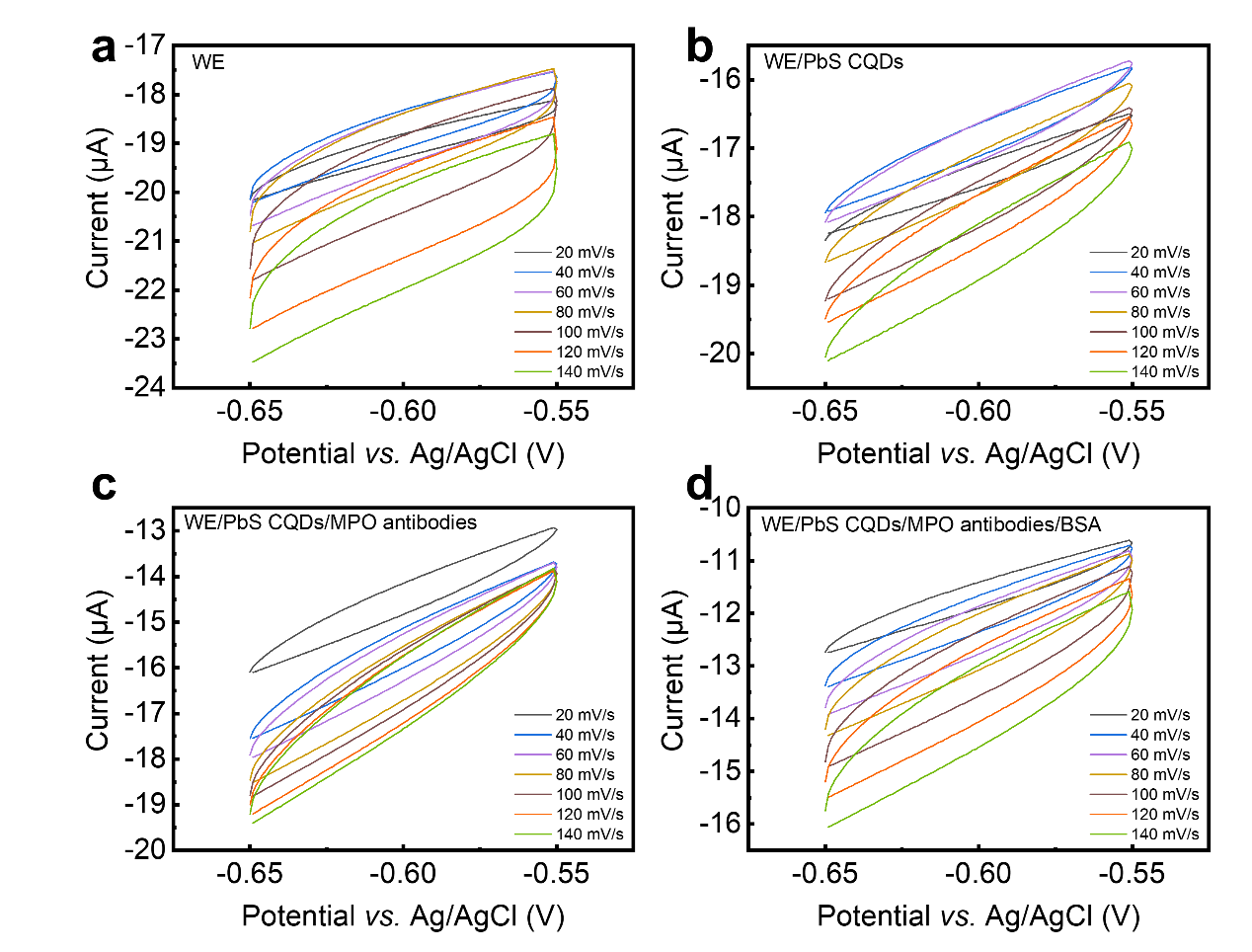


**Fig.** S6 The cyclic voltammetry curves of different modified electrodes at various scan rates in the non-faradaic region: (a) WE; (b) WE/PbS CQDs; (c) WE/PbS CQDs/MPO antibodies; (d) WE/PbS CQDs/MPO antibodies/BSA.


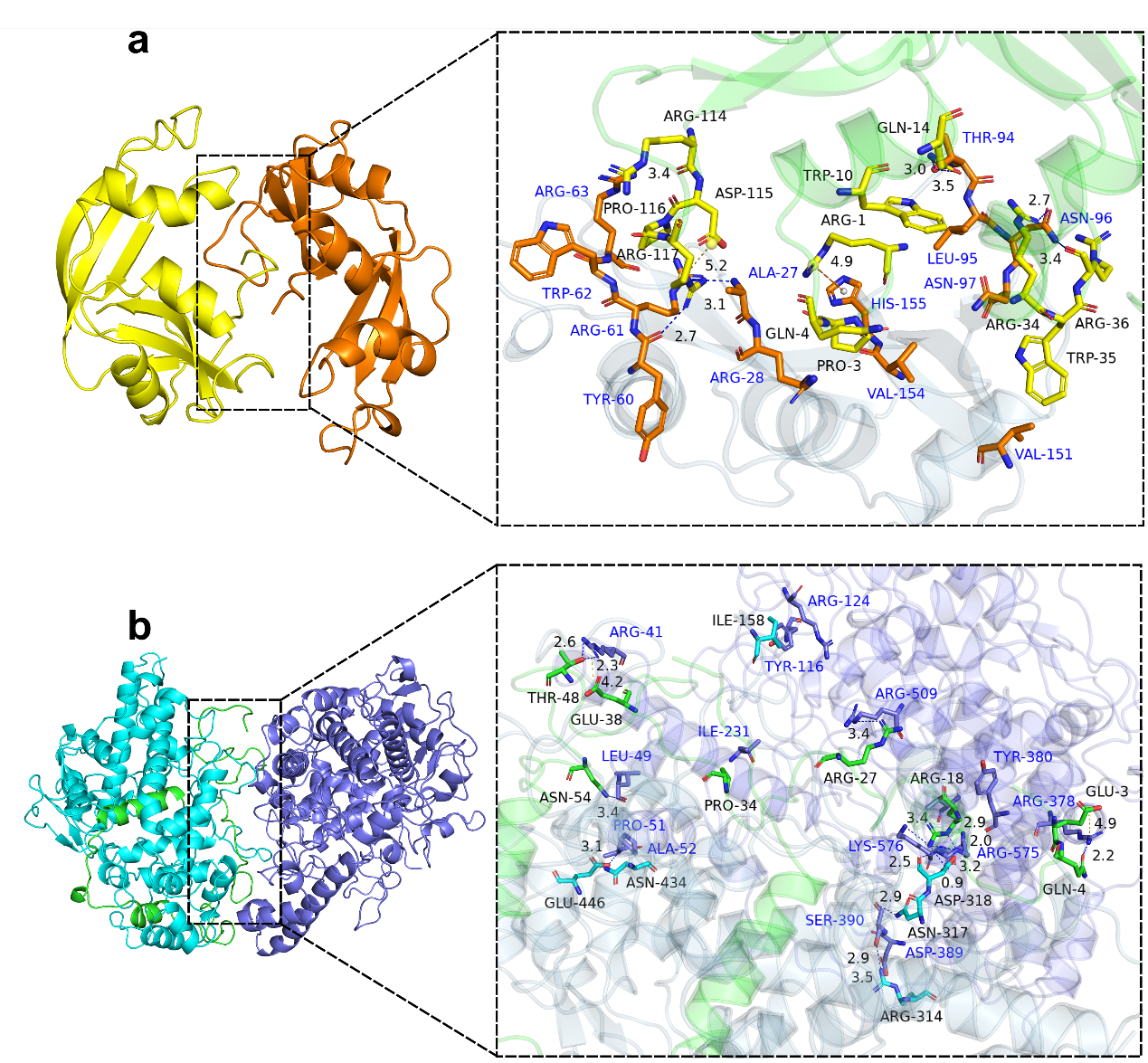


**Fig.** S7 Interaction between antigen and antibody: (a) ECP (yellow) and ECP antibody (orange). (b) MPO (green for chain A and light cyan for chain C) and MPO antibody (purple). The blue dashed line, yellow dashed line, and orange dashed line represent hydrogen bonds, salt bridges, and pi-cation interactions, respectively.


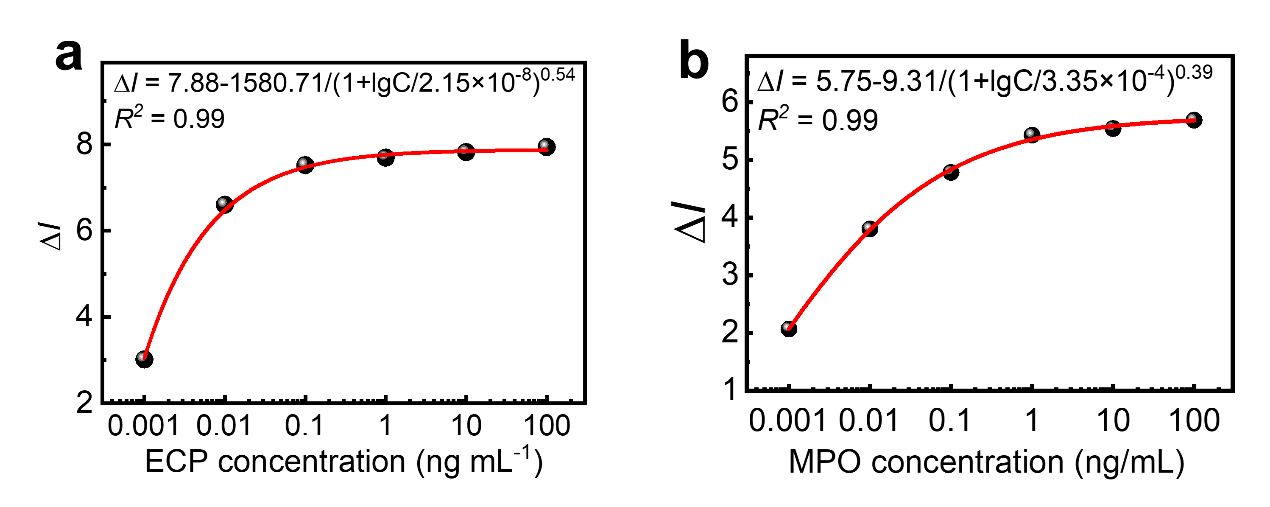


**Fig.** S8 (a) ∆*I*-Concentration Curve of ECP Standards. (b) ∆*I*-Concentration Curve of MPO Standards. Response (∆*I*) is defined as the ratio of the DPV peak current of the standard to that of the blank sample.

**Table.** S1 Detection performance of some reported ECP/MPO sensors.

| **Materials** | **Methods** | **Target** | **Detection Range** | **Ref** |
| --- | --- | --- | --- | --- |
| Hep-Au@Fe_3_O_4_/Ru(NH_3_)_6_^3+^ | Electrochemical | ECP | 1 - 1000 nM | [3] |
| CrRNA | CRISPR/Cas12a | MPO | 2 - 600 ng mL^-1^ | [4] |
| MWCNT/TMB/Au NPs | Electrochemical | MPO | 5 - 300 ng mL^-1^ | [5] |
| graphene-coated IDE-arrays/scFv | Electrochemical | MPO | / | [6] |
| Au NPs/poly(o-phenylenediamine)/MWCN | Electrochemical | MPO | 0.2 - 23.4 ng mL^−1^  /23.4 - 300 ng mL^−1^ | [7] |
| nanogold/cerium dioxide-BMIMPF_6_/l-Cysteine | Electrochemical | MPO | 10 - 400 ng mL^-1^ | [8] |
| GNPs/GR/ferricyanide | Electrochemical | ECP | 0.01 - 10 μM | [9] |
| PbS CQDs/Antibodies/BSA | Electrochemical | ECP/MPO | 0.001 - 100 ng mL^-1^ | This work |

[3] C.-Y. Lee, L.-P. Wu, T.-T. Chou, Y.-Z. Hsieh, Functional magnetic nanoparticles–assisted electrochemical biosensor for eosinophil cationic protein in cell culture, Sensors and Actuators B: Chemical 257 (2018) 672-677.

[4] J.-Y. Ma, B. Liu, S. Raza, H.-X. Jiang, A.-N. Tang, D.-M. Kong, CRISPR/Cas12a-based hypochlorous acid and myeloperoxidase biosensors designed on RESET effect, Sensors and Actuators B: Chemical 376 (2023) 133000.

[5] R. Nandeshwar, S. Tallur, Electrochemical detection of myeloperoxidase (MPO) in blood plasma with surface-modified electroless nickel immersion gold (ENIG) printed circuit board (PCB) electrodes, Biosensors and Bioelectronics 246 (2024) 115891.

[6] L.E. Delle, V. Pachauri, S. Sharma, O. Shaforost, H. Ma, M. Adabi, R. Lilischkis, P. Wagner, R. Thoelen, N. Klein, R. O’Kennedy, S. Ingebrandt, ScFv-modified graphene-coated IDE-arrays for ‘label-free’ screening of cardiovascular disease biomarkers in physiological saline, Biosensors and Bioelectronics 102 (2018) 574-581.

[7] B. Liu, L. Lu, Q. Li, G. Xie, Disposable electrochemical immunosensor for myeloperoxidase based on the indium tin oxide electrode modified with an ionic liquid composite film containing gold nanoparticles, poly(o-phenylenediamine) and carbon nanotubes, Microchimica Acta 173 (2011) 513-520.

[8] L. Lu, B. Liu, S. Li, W. Zhang, G. Xie, Improved electrochemical immunosensor for myeloperoxidase in human serum based on nanogold/cerium dioxide-BMIMPF6/l-Cysteine composite film, Colloids and Surfaces B: Biointerfaces 86 (2011) 339-344.

[9] Z. Wang, J. Yang, L. Gui, Development of a Graphene-Based Aptamer Sensor for Electrochemical Detection of Serum ECP Levels, International Journal of Electrochemical Science 12 (2017) 9502-9511.

**Table. S**2 ECP/MPO detection rates of PbS CQDs-based immunosensorin different hemolysis conditions

|  | Hemolysis condition | | |
| --- | --- | --- | --- |
|  | (+) | (++) | (+++) |
| ECP detection rate | 99.998% | 100.003% | 99.999% |
| MPO detection rate | 99.997% | 99.981% | 99.986% |

**Table. S**3 ECP/MPO detection rates of PbS CQDs-based immunosensors for serum samples with different placement duration at RT

|  | 1 h | 2 h | 3 h | 4 h |
| --- | --- | --- | --- | --- |
| ECP detection rate | 49.500% | 49.259% | 24.921% | 25.838% |
| MPO detection rate | 94.515% | 73.203% | 71.124% | 70.557% |

**Table.** S4 ECP/MPO detection rates when serum samples stored at 4 ℃

|  | Day 1 | Day 3 | Day 5 | Day 7 |
| --- | --- | --- | --- | --- |
| ECP detection rate | 87.971% | 80.893% | 44.570% | 46.403% |
| MPO detection rate | 99.592% | 92.580% | 95.634% | 91.161% |

**Table.** S5 ECP/MPO detection rates when serum samples stored at -20 ℃

|  | Day 1 | Day 3 | Day 5 | Day 7 |
| --- | --- | --- | --- | --- |
| ECP detection rate | 97.784% | 99.971% | 101.209% | 101.311% |
| MPO detection rate | 99.942% | 98.731% | 100.101% | 97.662% |

**Table.** S6 Results of spike recovery experiment in serum samples using PbS CQDs/ECP antibodies immunosensors

| Samples | $T$  (ng mL^-1^) | $C_{balnk/spiked}$  (ng mL^-1^) | | $SD$  (n=3) | $\text{CV}\text{ }$  (%, n=3) | $\text{P}$  (%) |
| --- | --- | --- | --- | --- | --- | --- |
| Serum sample I  ECP: 10 ng mL^-1^  MPO: 0 ng mL^-1^ | 0.00 | | 0.08 | / | / | / |
|  | 1.00 | | 1.09 | 0.13 | 11.93 | 101.00 |
|  | 10.00 | | 10.22 | 0.43 | 4.21 | 101.40 |
|  | 100.00 | | 103.20 | 3.52 | 3.41 | 103.12 |
| Serum sample II  ECP: 0 ng mL^-1^  MPO: 10 ng mL^-1^ | 0.00 | | 0.02 | / | / | / |
|  | 1.00 | | 1.08 | 0.10 | 9.26 | 106.00 |
|  | 10.00 | | 10.28 | 0.93 | 9.05 | 102.60 |
|  | 100.00 | | 105.60 | 4.22 | 4.00 | 105.58 |
| Serum sample III  ECP: 10 ng mL^-1^  MPO: 10 ng mL^-1^ | 0.00 | | 0.09 | / | / | / |
|  | 1.00 | | 1.20 | 0.21 | 17.50 | 111.00 |
|  | 10.00 | | 8.98 | 0.49 | 5.46 | 88.90 |
|  | 100.00 | | 95.30 | 4.21 | 4.42 | 95.21 |

**Table.** S7 Results of spike recovery experiment in serum samples using PbS CQDs/MPO antibodies immunosensors

| Samples | $T$  (ng mL^-1^) | $D_{balnk/spiked}$  (ng mL^-1^) | | $SD$  (n=3) | $\text{CV}\text{ }$  (%, n=3) | $\text{P}$  (%) |
| --- | --- | --- | --- | --- | --- | --- |
| Serum sample I  ECP: 10 ng mL^-1^  MPO: 0 ng mL^-1^ | 0.00 | | 0.03 | / | / | / |
|  | 1.00 | | 0.99 | 0.02 | 2.02 | 96.00 |
|  | 10.00 | | 9.89 | 0.92 | 9.30 | 98.60 |
|  | 100.00 | | 101.97 | 6.74 | 6.61 | 101.94 |
| Serum sample II  ECP: 0 ng mL^-1^  MPO: 10 ng mL^-1^ | 0.00 | | 0.09 | / | / | / |
|  | 1.00 | | 1.12 | 0.10 | 8.93 | 103.00 |
|  | 10.00 | | 9.86 | 0.94 | 9.53 | 97.70 |
|  | 100.00 | | 107.70 | 5.87 | 5.45 | 107.61 |
| Serum sample III  ECP: 10 ng mL^-1^  MPO: 10 ng mL^-1^ | 0.00 | | 0.07 | / | / | / |
|  | 1.00 | | 1.20 | 0.10 | 8.33 | 113.00 |
|  | 10.00 | | 9.95 | 0.45 | 4.52 | 98.80 |
|  | 100.00 | | 102.80 | 3.77 | 3.67 | 102.73 |

**Table.** S8 Detection results of PbS CQDs-based immunosensors in nasal secretion samples

| Number | response | Sensors  (ECP)  (ng mL^-1^) | ELISA  (ECP)  (ng mL^-1^) | Number | response | Sensors  (MPO)  (ng mL^-1^) | ELISA  (ECP)  (ng mL^-1^) |
| --- | --- | --- | --- | --- | --- | --- | --- |
| 1 | 2.12 | 200.40 | 227.21 | 1 | 1.86 | 300.50 | 323.72 |
| 2 | 2.08 | 192.09 | 214.84 | 2 | 1.64 | 210.92 | 248.00 |
| 3 | 2.06 | 188.32 | 197.32 | 3 | 1.57 | 178.01 | 210.15 |
| 4 | 1.86 | 150.88 | 166.96 | 4 | 1.53 | 160.90 | 172.06 |
| 5 | 1.83 | 144.30 | 159.06 | 5 | 1.59 | 189.38 | 158.22 |
| 6 | 1.82 | 141.92 | 153.14 | 6 | 1.51 | 155.07 | 147.74 |
| 7 | 1.85 | 148.33 | 124.43 | 7 | 1.38 | 98.59 | 144.59 |
| 8 | 1.75 | 128.27 | 117.14 | 8 | 1.44 | 126.02 | 108.60 |
| 9 | 1.58 | 95.61 | 112.98 | 9 | 1.42 | 115.37 | 108.31 |
| 10 | 1.50 | 80.52 | 92.69 | 10 | 1.38 | 98.02 | 103.49 |
| 11 | 1.45 | 72.01 | 78.76 | 11 | 1.34 | 82.45 | 91.65 |
| 12 | 1.44 | 68.49 | 77.54 | 12 | 1.36 | 89.23 | 81.99 |
| 13 | 1.47 | 75.28 | 68.16 | 13 | 1.31 | 70.16 | 81.88 |
| 14 | 1.40 | 62.21 | 68.14 | 14 | 1.35 | 87.85 | 81.88 |
| 15 | 1.40 | 62.01 | 64.19 | 15 | 1.31 | 70.04 | 76.09 |
| 16 | 1.44 | 70.09 | 57.48 | 16 | 1.33 | 76.38 | 60.30 |
| 17 | 1.49 | 78.71 | 56.09 | 17 | 1.35 | 88.29 | 47.88 |
| 18 | 1.34 | 50.13 | 55.95 | 18 | 1.26 | 48.87 | 36.24 |
| 19 | 1.25 | 31.86 | 43.80 | 19 | 1.25 | 46.40 | 34.92 |
| 20 | 1.43 | 67.32 | 42.46 | 20 | 1.19 | 20.91 | 34.63 |
